# Bisphenol A, Tobacco Smoke, and Age as Predictors of Oxidative Stress in Children and Adolescents

**DOI:** 10.3390/ijerph16112025

**Published:** 2019-06-06

**Authors:** Roberto Bono, Valeria Bellisario, Roberta Tassinari, Giulia Squillacioti, Tilde Manetta, Massimiliano Bugiani, Enrica Migliore, Pavilio Piccioni

**Affiliations:** 1 Department of Public Health and Pediatrics, University of Turin, 10126 Turin, Italy; valeria.bellisario@unito.it (V.B.); roberta.tassinari@unito.it (R.T.); giulia.squillacioti@unito.it (G.S.); tilde.manetta@unito.it (T.M.); 2 Consultant of OMP (observatory of professional diseases) of the Turin Court Prosecutor’s Office, Turin 10100, Italy; maxbugiani@fastwebnet.it; 3 Cancer Epidemiology, AOU Città della Salute e della Scienza di Torino, Turin 10126, Italy; enrica.migliore@unito.it; 4 Unit of Pneumology and Tisiology, National Health Service (ASL TO2), Torino 10100, Italy; papiccioni@gmail.com

**Keywords:** oxidative stress, adolescents, passive tobacco smoke, BPA, public health

## Abstract

Objectives. The purpose of this study was to investigate bisphenol A (BPA) and its role in the induction of oxidative stress and confirm the same for tobacco smoke. Methods. A total of 223 young, healthy students (7–19 years old) were recruited in Chivasso, Italy. A spot of urine of each subject was analyzed to quantify BPA, cotinine, and 15F2t-isoprostane. Results. BPA showed a slight increase of concentration proportional with increasing age, even though the 11–14 years age group had slightly lower results, inducing a V-shape. The same trend was observed for 15F2t-isoprostane and cotinine. The result of piecewise linear robust regression shows a break point of the effect of BPA on 15F2t-isoprostane at 6 ng/mg CREA (*p* < 0.001). At higher levels, 15F2t-isoprostane shows an exponential increase by more than threefold for each one-log unit of BPA. An increase of oxidative stress due to BPA was observed, but only from 6 ng/mg of CREA up. Passive tobacco smoke is also able to induce an increase in oxidative stress. Conclusion. Prevention against BPA and passive tobacco smoke represents an important tool for promoting the highest health standard.

## 1. Introduction

Due to its endocrine disruptor properties and widespread presence in the human life environment, bisphenol A (BPA) is an important topic in terms of public health. BPA, whose IUPAC (International Union of Pure and Applied Chemistry) name is 2, 2-bis (4-hydroxyphenyl) propane (CASRN: 80-05-7), is a synthetic organic compound with a relatively short life [1]. The monomeric form of BPA is used in plastic food contact materials, in accordance with Commission Regulation (EU) No. 10/2011/EU on plastic materials coming into contact with foodstuffs. Furthermore, based on the precautionary principle, in 2011, the European Commission introduced the Implementing Regulation (EU) No 321/20118, which placed a restriction on the use of BPA in the manufacture of infant feeding bottles. 

According to the European Food Safety Authority (EFSA), the general population can be exposed to BPA in external, internal, and aggregated ways via food, dermal contact (cosmetics and thermal paper), drinking water, swimming, and/or breathing indoor and outdoor air [2]. However, breast milk represents the main vehicle of human intake of BPA, which determines its highest concentrations in the urine of young children [3]. 

Although BPA is not dangerous in its polymeric form, its transformation in the monomeric form can be realized in acidic or basic solutions and when exposed to UV light. Thus, over time, food and drink containers can become a widespread public health risk [4]. Furthermore, the negative effects of BPA can be evident for children and adolescents, pregnant women, and their embryos, as confirmed by numerous tests on animals in vivo and in vitro [5,6]. Nevertheless, only free (unconjugated) BPA is a weak estrogen [7], and its presence in the different biological matrices is substantially negligible. This is due to an efficient metabolization of BPA together with a biological half-life in humans of less than six hours [8,9]. 

BPA, as an endocrine disruptor, is able to contribute to or induce several other negative effects, including reproductive, perinatal, and pediatric outcomes, hepatic tumors, lung inflammation, Parkinson disease, abnormal behavior, obesity, diabetes, and reproductive abnormalities in offspring [10]. Furthermore, BPA is able to induce an increase in oxidative stress [11,12,13].

Usually, BPA is detectable in urine, blood, breast milk, semen, cord blood, fetal serum, placental tissue, and animal fat [14,15], but in urine, its detection frequency is about 75–90% [16,17]. Glucuronic acid of BPA (GlcA–BPA) is also an urinary metabolite of BPA, and it is currently considered the major residue of BPA, both in vitro and in vivo [18], which makes it suitable for molecular epidemiology studies. BPA contributes to lipid peroxidation (LPO), and therefore, as mentioned earlier, to the induction of oxidative stress (OS), which is a biological imbalance that occurs when endogenous and/or exogenous oxidants overtake the level of antioxidant defenses [19,20,21]. 

The urinary BPA in children is significantly more concentrated than in adults because they eat, drink, and breathe in greater quantities per kilogram of body weight [15,22]. Furthermore, children are more sensitive and fragile because their metabolism system and organs are not yet fully developed [23]. In particular, infants up to two or three months of age might have higher free-BPA levels in urine since detoxifying enzymes such as UDP-glucuronosyltransferase are not yet fully developed [2,24]. Due to the widespread exposure to BPA and the consequent potential health risk to humans, restrictions and dedicated regulations for the use of this toxic chemical have been suggested worldwide. In 2015, the EFSA [25] reduced the temporary Tolerable Daily Intake (t-TDI) of BPA from 50 to 4 µg/kg bw/day. Consequently, BPA is being replaced with a number of alternatives.

Although the presence of oxidative stress is a known prepatological condition of numerous health effects, including atherosclerosis, cardiovascular disease, cancer, and pregnancy outcomes [26], currently, only a few studies on adults, and very few on children have explored the exposure to BPA in relation to the induction of inflammation, LPO, and OS [27,28]. Thus, the aim of the present study has been to investigate the presence of BPA in the urine of a group of adolescents, its role in the induction of OS, and to confirm the same role of tobacco smoke [29,30,31]. Furthermore, given that our previous works had shown an unexpectedly decreasing trend in oxidative stress among adolescents, in this work, we wanted to check this contrasting trend again with the other life phases.

To achieve this goal, a sample of urine provided by every one of the 223 young healthy volunteers (7–19 years old) attending three different schools of Chivasso (close to Torino, Piedmont, northwestern Italy) was analyzed to quantify BPA, cotinine, and 15F2t-Isoprostane (15F2t-IsoP). The first was a chemical directly detectable in urine as an internal dose biomarker, the second was a nicotine metabolite to quantify exposure to smoking (an internal dose biomarker, too), and the third was a biomarker of OS. We chose 15F2t-IsoP because it is one of the most stable, sensitive, and non-invasive biomarkers of oxidative stress in urine; this is because it is a specific and stable product of lipid peroxidation that is largely used for in vivo investigations. [32]. 

## 2. Materials and Methods

### 2.1. Selection of Subjects

All the 223 students who voluntarily participated to this study attended three different schools at Chivasso, which is a medium urbanized town with about 27,000 inhabitants (522 inhabitants/km^2^) located at 180 m above sea level close to Torino (the metropolitan city of the Piedmont Region, Italy—890,500 inhabitants). No other selection criteria were adopted to recruit volunteers. Since the subjects were underage, parents and teachers were informed during a public meeting on the objective of this study, and consequently, written informed consent was signed and delivered by each participants’ parents. Moreover, the participation of all the subjects took place only after obtaining the assent of the local Ethics committee of “San Luigi” Turin Hospital (session on 11 March 2015 authorization number 27/2015). Samplings were carried out from January to March, involving one class per day, on Wednesday or Thursday, according to a pre-established timetable. A questionnaire was administered, and a urine sample was collected from each student. 

### 2.2. Questionnaire

To each subject, one interviewer administered a questionnaire during school hours. The answers provided information on individual and clinical features, such as age, weight, and height, gender, residence, diet (dinner the day before), hobbies, therapies, and health conditions. The questionnaire used was mainly a synthesis of the most extensive questionnaire “SIDRIA”, which has been described in detail elsewhere [33].

### 2.3. Urine

A spot of urine was collected from each volunteer during the morning sampling to measure the following parameters:

#### 2.3.1. BPA

To exclude contamination from BPA, all the urine samples were collected in BPA-free plastic vessels (polypropylene) and stored at −80 °C until analysis. All the laboratory glass material that was used was washed with methanol and then kept in methanol for 12 hours, which was subsequently analyzed to verify the possible contamination of BPA. Each thawed sample of urine was vortexed, and 700 µL of acetonitrile, 750 µL of ethyl acetate, and 10 µL of BPA-d_16_ (1 ng/µL), which were used as internal standards, were added to each 400-µL urine sample. To facilitate the liquid–liquid extraction (LLE), samples were vortexed for 3 minutes; then, they were centrifuged at 4000 rpm for 15 min, and the supernatants were evaporated to dryness by a gentle stream of nitrogen. The dried extract was dissolved with 125 µL of methanol/water (1:1 *v/v*) and analyzed by HPLC—MS/MS to quantify GlcA–BPA. GlcA–BPA was identified and quantified by liquid chromatography equipped with a low-pH resistant reverse phase column, Kinetex EVO C18 (2.6 μm, 150 × 3.0 mm). The binary solvent system was: (a) acidified ultrapure water with formic acid 0.1% *v/v* and (b) acetonitrile (HPLC ultrapure grade) acidified with formic acid 0.1% *v/v*. The chromatographic separation was carried out at constant flow rate (200 µL/min^−1^) and constant temperature (23 °C ± 1 °C) by a column thermostat. The solvent linear gradient was from 10% to 30% of B in 5 min, to 65% of B at 30 min, and 95% of B at 33 min. The concentration of solvent B was maintained at 95% for 5 min. The initial mobile phase was re-established for 10 min before the next injection. The injection volume was 20 μL, and quantification was performed by internal standard method (BPA-d_16_). Quantitative analyses were carried out by tandem mass spectrometry with a 6330 Series Ion Trap LC-MS system equipped with an electrospray ionization source (ESI). The analytes were detected in negative mode. The dry gas (Nitrogen) was at 325 °C, 20.0 psi, and 10 L min^−1^; capillary voltage was at 2000 V. Data acquisition was made in multiple reaction monitoring (MRM) mode by monitoring the transitions of quasi-molecular ions [M-H]: 227 for BPA, 242 for BPA-d_16_, 307 for HO_3_S–BPA, 403 for GlcA–BPA, and 419 for OH–GlcA–BPA. Procedural blank samples with ultrapure water in the place of urine were collected, extracted, and analyzed by HPLC-MS/MS with the same sample protocol. In the processed blanks, BPA contaminations above the limit of detection (LOD, 0.065 ng·mL^−1^) were not detected.

#### 2.3.2. Cotinine

Urine samples were prepared for analysis as follows: 10 ml of urine were fortified with 10 µL of cotinine-d_3_ as an internal standard, 4 g of NaCl, and 500 µL of NaOH (5 M). Then, 2 mL of CHCl_3_ was added two times to extract the cotinine by means of LLE for 15 min. Then, each sample was centrifuged for 10 min at 1000× *g*, and the resulting organic phase was collected in a glass tube and evaporated to dryness in a rotary evaporator at room temperature. The dry residue was reconstituted in 200 µL of CHCl_3_ and transferred into a conical vial for GC-MS determination [34].

#### 2.3.3. 15.F_2t_-Isoprostane (15F_2t_-IsoP)

15.F_2t_-IsoP was measured to quantify OS by the ELISA technique, which was carried out with a specific microplate kit (Oxford, MI, USA) and according to the manufacturer’s instructions. To achieve better accuracy in the competitive ELISA method, each sample was diluted 1:4. Our previous paper reports all the details of this procedure [32]. 

#### 2.3.4. Creatinine

In order to normalize the excretion rate of cotinine, 15F_2t_-IsoP, GlcA–BPA, and an aliquot of fresh urine were used to quantify the concentration of creatinine (CREA) by the kinetic Jaffè procedure.

### 2.4. Statistical Analysis

Statistical analysis was performed by means of Stata 12 Statistical Package (Stata Corp LP, Lakeway Drive, TX, USA). Appropriate linear transformation was applied on data whenever suggested by distributional diagnostic plots (symmetry plot, quantile plot) and descriptive statistic inspection (looking at variance stability among categories). 

In inspecting the two-way plot of log (ng 15F_2t_-IsoP/mg CREA) versus log (GlcA–BPA), a non-linear relationship between these variables was detected, suggesting a threshold value of the (GlcA–BPA) on (ng 15F2t-IsoP/mg CREA). So, to estimate a spline function, we used piecewise linear or “hockey stick” robust multiple regression [35] using Box–Cox transformed ng 15F2t-IsoP/mg CREA as the dependent and Box–Cox transformed (GlcA–BPA). This presupposes that two straight lines, with different slopes, and calculating the two slopes and the value of the dependent at which the slope changes (the breakpoint or spline point), can best fit the effect of predictive variables on dependents.

In the model log (ng cotinine/mg CREA), the effects of linear body mass index (BMI), gender, and age classes were also tested and retained in the model as covariates when the 5% significance of the effect was reached or significantly changed the estimates. 

## 3. Results

In Table 1, the characteristics of students enrolled for the study are reported. Numerousness, mean, standard deviation (s.d.), and percentage (%) for gender, age (years), height (m), weight (kg), and smoking exposure (number of cigarettes per day) are shown for the subjects grouped for educational level. Among the 223 students, 18 reported being active smokers (8%), which were all from the 14–19 age group; 52 were passive smokers (23.3%), and 153 were non-smokers (68.7%). In Table 2, cotinine, 15F_2t_-IsoP, and GlcA–BPA—all expressed as nanograms per 1 milligram of creatinine—are listed according to educational level as mean, standard deviation, minimum, and maximum. 

**Figure 1:**
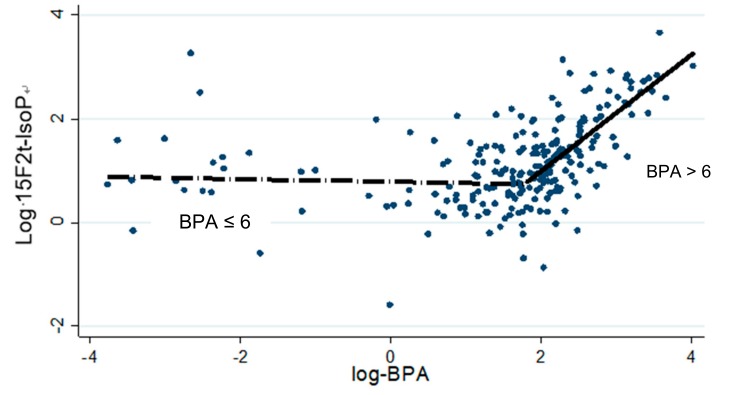
Piecewise linear robust regression of the relation of log glucuronic acid of bisphenol A (GlcA–BPA) on log (ng 15F_2t_-IsoP/mg CREA)—(break point at BPA = 6 ng/mg creatinine (CREA), 95% CI: 4.5—7.5). Exp (1.79) = 6.

**Figure 2:**
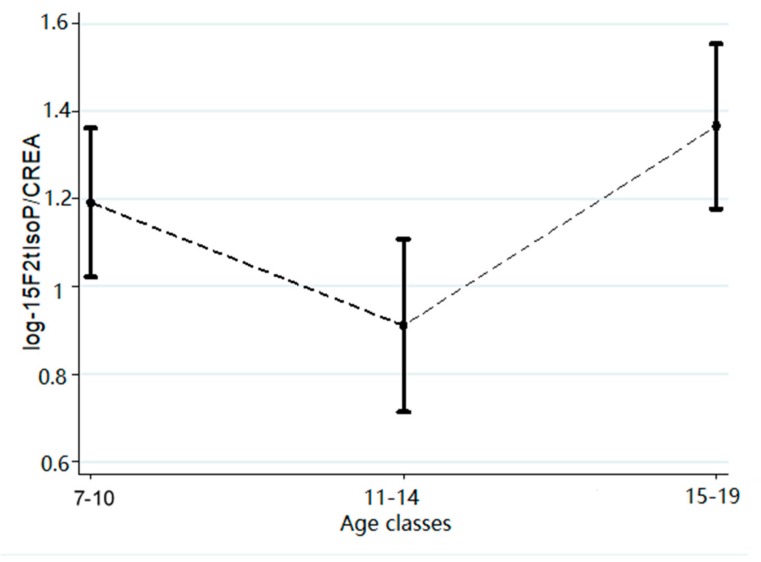
Margins plot of the relation between log 15F2t–IsoP and age classes.

GlcA–BPA shows an increase of concentration proportional with increasing age, even if the intermediate age group (11–14 years) is slightly lower. The same thing is observed also for 15F_2t_-IsoP and the exposure to tobacco (mainly passively breathed) quantified by cotinine. According to the Box–Cox regression results, the values of the biological markers analyzed were subjected to a logarithmic transformation before carrying out the subsequent analysis. The result of piecewise linear robust regression shows a breakpoint at 1.79 (95% CI: 1.56–2.02; *p* < 0.001) of the effect of log-GlcA–BPA on log-15F_2t_-IsoP (Figure 1 and Table 3). Thus, the concentration of 15F_2t_-IsoP increases exponentially (more than threefold for each one-log unit of GlcA–BPA), when the log-GlcA–BPA concentration overcomes the breakpoint identified at 1.79 log-GlcA–BPA (6 ng/mg CREA). Multiple Linear Regression (MLR) analysis shows a positive effect also of log cotinine concentration on log 15F_2t_-IsoP (Table 3). This last effect is evident even considering that a 12% increase of 15F_2t_-IsoP is observed for each increment of a log-cotinine unit. Furthermore, the analysis of the relationship between log (ng 15F_2t_-IsoP/mg CREA) and age shows a V-shaped trend (Figure 2), with a significant decrease (*p* = 0.026) between infancy (7–10 years old) and the beginning of adolescence (11–15 years old), and then a new increase starting from 15 years of age (Figure 2 and Table 4). 

## 4. Discussion

The main objective of this work was to evaluate the environmental diffusion and the possible consequent absorption of BPA in a population of children and adolescents attending primary, secondary, and high school in a city located in Piedmont region, in the northwestern part of Italy. At the same time, we wanted to observe the role of this pollutant in the induction of OS, taking into account as confounders, the role of passive and active exposure to tobacco smoke and age, and other predictors of the same effect. These youth were enrolled as a population that is useful for investigating some environmental conditions as predictors of OS status development as accurately as possible. This is because their life habits lead them to be more in contact with the outside environment and because their lower body weight makes them more sensitive and vulnerable. Regarding this concern, it is also known that young people are still in a phase of development of the body and of their metabolic system, and therefore still fragile and hypersensitive to environmental stimuli.

The OS level was monitored through the quantification of urinary 15F_2t_-IsoP concentration, which is a biomarker that is unaffected by diet, potentially confounding the relationship we have investigated [36,37]. Furthermore, the diet was very similar among all the students. This was known from the replies to the questionnaire—they outlined a homogeneous domestic diet—and because they benefit from the same school lunch prepared by the same company according to the requirements imposed by nutritionists working at the local health authority to minimize oxidant food.

Since the exposure to BPA can influence the OS level, urinary GlcA–BPA was measured to understand the role of this contaminant in the onset of 15F_2t_-IsoP values. The findings show that the effect of log GlcA–BPA on 15F_2t_-IsoP has a threshold value around a breakpoint of 1.79. This suggest that values of GlcA–BPA lower than 4.5 ng/mg of creatinine (exponential value of lower confidential limit) have no measurable effect on isoprostane; conversely, above the breakpoint (6 ng/mg crea), 15F_2t_-IsoP grows linearly (*p* < 0.005). To explain this log-linear relationship characterized by a threshold value, we have to remember the higher commitment of the liver to contrast the higher concentrations of this contaminant, or an insufficient sensitivity of analytical technique to detect BPA at lower concentrations. Nevertheless, this last hypothesis seems to be contradicted by the log-linear relationship without the threshold of the 15F_2t_-IsoP value versus cotinine. Indeed, the induction of oxidative stress by passive and/or active smoking was confirmed in adolescent subjects independently from age, which was also in our previous paper [38]. 

The age of the subject proved to be another factor that can significantly influence the 15F_2t_-IsoP concentration. In a previous work [38], the 15F_2t_-IsoP levels were studied in the 11–15 age group. A slight decrease (6%) was recorded when passing from 11 to 15 years. In the present study, the analysis of 15F_2t_-IsoP levels according to age (7–19 years old) highlighted the V-shape previously illustrated. This seems to confirm that the OS experiences a lowering of intensity in the first years considered, and then return to grow regularly. This may result in the establishment and growth of a condition of chronic inflammation until senescence [37,39,40].

Finally, we found that urine GlcA–BPA concentrations were positively but not significantly associated with BMI. Due to its rapid metabolism (half-life less than 6 h), BPA exposure estimates from first morning urine may just represent the exposure at the prior meal (dinner), rather than daily or average exposure level. Given the food indigestion as the main exposure route to BPA, perhaps more urine samples should be collected throughout the day preceding the sampling to avoid the underestimation of exposure to this contaminant. 

We can conclude that the adolescents studied showed an increase in OS dependent from GlcA–BPA higher than 4.8 ng/mg CREA, and from tobacco smoke passively and/or actively breathed. The induction of oxidative stress by GlcA–BPA is a theme that has not yet been analyzed in depth by the International Scientific Community. The public health authorities must consider it in a careful manner and without forgetting the other bisphenols that are now present in the living environment. Thus, the evidence of these risky conditions for public health may represent a platform for designing new preventive strategies addressed at promoting adolescent health in a sensitive period of growth, sexual differentiation, and brain development. Therefore, further studies on new and safer materials that have the least impact on the environment and human health are crucial. 

The main results obtained in this work are: GlcA–BPA causes an increase in OS in the adolescents selected for the study, but only starting from 6 ng/mg of CREA. In addition, the passively breathed tobacco smoke is able to induce an increase of the OS. Therefore, the promotion of health must also consist of the preventive contrast to BPA and all the bisphenols still present in the living environment.

## 5. Limitations and Future Purposes

A limitation of this study is that we planned a cross-sectional study design in different age ranges. Besides, our data had not been collected to specifically assess diet or other potential cofounders, such as environmental pollution. Instead, we intend to plan a longitudinal study to confirm all the trends found in this fist explorative research, both in terms of relationships between oxidative stress and BPA exposure and of possible roles of different confounding factors. 

## 6. Conclusions

Apart from the already demonstrated role of passive exposure to tobacco smoke [41], an increase of oxidative stress was observed also consequently to exposure to BPA, but only from 6 ng/mg of CREA upwards. In effect, 15F2t-isoprostane has proved to be positively correlated with exposure to BPA and tobacco smoke. This highlights the role of the risk factor of these pollutants in the increase of oxidative stress. Thus, the prevention and contrast regarding the exposure to BPA and passive tobacco smoke represent an important tool to promote the highest health standard in a category of subjects that is so particularly sensitive to the quality of the living environment.

## Figures and Tables

**Table 1:** Gender, age, height, weight, and number of active and passive smokers in the whole population and in three groups subgrouped according to the three educational level considered.

Characteristics of Students	Primary School (7–10 Years)	Secondary School (11–14 Years)	High School (15–19 Years)	Total
N.	87	34	102	223
Gender N. (%)	Male 47 (54.0%) Female 40	Male 15 (44.1%) Female 19	Male 57 (55.8%) Female 45	Male 119 (53.4%) Female 104
Age (years) Mean ± s.d.	8.87 ± 1.0	11.7 ± 0.8	16.6 ± 1.71	12.8 ± 3.8
Height (m) Mean ± s.d.	1.39 ± 0.08	1.54 ± 0.1	1.71 ± 0.08	1.56 ± 0.17
Weight (kg) Mean ± s.d.	35.6 ± 9.8	45.0 ± 7.5	64.5 ± 12.4	50.2 ± 17.2
Smoking habits N (%)	Active 0 Passive 26 (30%) Not exposed 61 (70%)	Active 0 Passive 5 (14.7%) Not exposed 29 (85.3%)	Active 18 (17.6%) Passive 21 (20.5%) Not exposed 63 (61.9%)	Active 18 (8%) Passive 52 (23.3%) Not exposed 153 (68.7%)

**Table 2:** Urinary cotinine, 15F2t-IsoP, and total BPA inactivated values in the three groups subgrouped according to the three educational level considered. g-Mean = geometric mean, s.d. = geometric standard deviation, Min = minimum value; Max = maximum value. Units of biological markers are nanograms of every 1 mg of urinary creatinine.

Educational Level	Cotinine [ng/mg CREA]	15F_2t_-IsoP [ng/mg CREA]	Total BPA Inactivated [ng/mg CREA]
	g-Mean (±s.d.) Min–Max	g-Mean (±s.d.) Min–Max	g-Mean (±sd) Min–Max
Primary school (7–10)	11.2 (±8.1) 1.06–382.9	3.3 (±2.2) 0.6–38.8	2.3 (±6.8) 0.02–38.7
Secondary school (11–14)	2.81 (±13.4) 0.1–372.3	2.5 (±2.1) 0.5–17.1	5.4 (±2.5) 0.9–34.4
High school (15–19)	26.3 (±16.8) 0.1–1730.9	3.9 (±2.4) 0.4–23.2	8.4 (±2.2) 0.3–55.4
Total g-mean (±s.d.) min–max	9.8 (±13.9) 0.03–1730	3.2 (±2.8) 0.41–38.8	4.9 (±4.2) 0.02–55.4

**Table 3:** Pricewise multiple non-linear regression parameters, with means and 95% confidence interval (CI), of log 15F_2t_-IsoP as the dependent variable and log (total inactive BPA), log cotinine, and age as predictors.

log 15F_2t_-IsoP	Coef.	95% CI Lower limit–Upper Limit		*p*
breakpoint	1.79	1.56	2.02	0.00
breakpoint	1.79	1.56	2.02	0.00
Log (total inactive BPA) < breakpoint	−0.01	−0.10	0.08	0.82
≥ breakpoint	1.11	0.87	1.34	0.00
Log Cotinine (ng/mg CREA)	0.03	0.00	0.06	0.05
<10	0			
Age class 11–14	−0.20	−0.41	0.00	0.05
≥ 15	−0.07	−0.27	0.14	0.53
Constant	0.73	0.59	0.87	0.00

**Table 4:** Estimated means of log 15F_2t_-IsoP by age class adjusted for log (total inactive BPA), log cotinine by means of piecewise non-linear regression.

Age Classes		Means	95%c CI Lower Limit–Upper Limit	*p* <
Age (years old)	<10	1.19	1.02–1.36	NS
	11–14	0.91	0.71–1.11	<0.05
	≥15	1.37	1.37–1.18	NS

## References

[1] Srivastava S., Gupta P., Chandolia A., Alam I. (2015). Bisphenol A: A threat to human health?. J. Environ. Health.

[2] Mielke H., Gundert-Remy U. (2009). Bisphenol A levels in blood depend on age and exposure. Toxicol. Lett..

[3] Lakind J.S., Naiman D.Q. (2011). Daily intake of bisphenol A and potential sources of exposure: 2005–2006 National Health and Nutrition Examination Survey. J. Exp. Sci. Environ. Epidemiol..

[4] Talsness C.E., Andrade A.J.M., Kuriyama S.N., Taylor J.A., vom Saal F.S. (2009). Components of plastic: Experimental studies in animals and relevance for human health. Philos. Trans. R Soc. Lond. B Biol. Sci..

[5] Goodman J.E., McConnell E.E., Sipes I.G., Witorsch R.J., Slayton T.M., Yu C.J., Lewis A.S., Rhomberg L.R. (2006). An updated weight of the evidence evaluation of reproductive and developmental effects of low doses of bisphenol A. Crit. Rev. Toxicol..

[6] Richter C.A., Birnbaum L.S., Farabollini F., Newbold R.R., Rubin B.S., Talsness C.E., Vandenbergh J.G., Walser-Kuntz D.R., vom Saal F.S. (2007). In vivo effects of bisphenol A in laboratory rodent studies. Reprod. Toxicol..

[7] Shimizu M., Ohta K., Matsumoto Y., Fukuoka M., Ohno Y., Ozawa S. (2002). Sulfation of bisphenol A abolished its estrogenicity based on proliferation and gene expression in human breast cancer MCF-7 cells. Toxicol. In Vitro.

[8] Fukata H., Miyagawa H., Yamazaki N., Mori C. (2006). Comparison of Elisa-and LC-MS-Based Methodologies for the Exposure Assessment of Bisphenol, A. Toxicol. Mech. Methods.

[9] Tsukioka T., Terasawa J., Sato S., Hatayama Y., Makino T., Nakazawa H. (2014). Development of analytical method for determining trace amounts of BPA in urine samples and estimation of exposure to BPA. J. Environ. Chem..

[10] Wang M., Rang O., Liu F., Xia W., Li Y., Zhang Y., Lu S., Xu S. (2018). A systematic review of metabolomics biomarkers for Bisphenol A exposure. Metabolomics.

[11] Gassman N.R. (2017). Induction of oxidative stress by bisphenol A and its pleiotropic effects. Environ. Mol. Mutagen..

[12] Wang Y.-X., Liu C., Shen Y., Wang Q., Pan A., Yang P., Chen Y.-J., Deng Y.-L., Lu Q., Cheng L.-M. (2019). Urinary levels of bisphenol A, F and S and markers of oxidative stress among healthy adult men: Variability and association analysis. Environ. Int..

[13] Qiu W., Chen J., Li Y., Chen Z., Jiang L., Yang M., Wu M. (2016). Oxidative stress and immune disturbance after long-term exposure to bisphenol A in juvenile common carp (Cyprinus carpio). Ecotoxicol. Environ. Saf..

[14] Geens T., Roosens L., Neels H., Covaci A. (2009). Assessment of human exposure to Bisphenol-A, Triclosan and Tetrabromobisphenol-A through indoor dust intake in Belgium. Chemosphere.

[15] Vandenberg L.N., Gerona R.R., Kannan K., Taylor J.A., van Breemen R.B., Dickenson C.A., Liao C., Yuan Y., Newbold R.R., Padmanabhan V. (2014). A round robin approach to the analysis of bisphenol a (BPA) in human blood samples. Environ. Health.

[16] Wilson N., Chuang J., Morgan M.K., Lordo R.A., Sheldon L.S. (2007). An observational study of the potential exposures of preschool children to pentachlorophenol, bisphenol-A, and nonylphenol at home and daycare. Environ. Res..

[17] Pirard C., Sagot C., Deville M., Dubois N., Charlier C. (2012). Urinary levels of bisphenol A, triclosan and 4-nonylphenol in a general Belgian population. Environ. Int..

[18] Dekant W., Völkel W. (2008). Human exposure to bisphenol A by biomonitoring: Methods, results and assessment of environmental exposures. Toxicol. Appl. Pharmacol..

[19] Kim S., Mun G.-I., Choi E., Kim M., Jeong J.S., Kang K.W., Jee S., Lim K.-M., Lee Y.-S. (2018). Submicromolar bisphenol A induces proliferation and DNA damage in human hepatocyte cell lines in vitro and in juvenile rats in vivo. Food Chem. Toxicol..

[20] Bono R., Romanazzi V., Munnia A., Piro S., Allione A., Ricceri F., Guarrera S., Pignata C., Matullo G., Wang P. (2010). Malondialdehyde-deoxyguanosine adduct formation in workers of pathology wards: The role of air formaldehyde exposure. Chem. Res. Toxicol..

[21] Bono R., Romanazzi V. (2015). Isoprostanes as Biomarkers of Disease and Early Biological Effect. General Methods in Biomarker Research and Their Applications.

[22] Calafat A.M., Ye X., Wong L.-Y., Reidy J.A., Needham L.L. (2008). Exposure of the U.S. population to bisphenol A and 4-tertiary-octylphenol: 2003–2004. Environ. Health Perspect..

[23] Preuss R., Angerer J., Drexler H. (2003). Naphthalene? An environmental and occupational toxicant. Int. Arch. Occup. Environ. Health.

[24] Edginton A.N., Ritter L. (2009). Predicting plasma concentrations of bisphenol A in children younger than 2 years of age after typical feeding schedules, using a physiologically based toxicokinetic model. Environ. Health Perspect..

[25] EFSA (2015). Report on the Two-Phase Public Consultation on the Draft EFSA Scientific Opinion on Bisphenol A (BPA).

[26] Watkins D.J., Ferguson K.K., Anzalota Del Toro L.V., Alshawabkeh A.N., Cordero J.F., Meeker J.D. (2014). Associations between urinary phenol and paraben concentrations and markers of oxidative stress and inflammation among pregnant women in Puerto Rico. Int. J. Hyg. Environ. Health.

[27] Hong Y.-C., Park E.-Y., Park M.-S., Ko J.A., Oh S.-Y., Kim H., Lee K.-H., Leem J.-H., Ha E.-H. (2009). Community level exposure to chemicals and oxidative stress in adult population. Toxicol. Lett..

[28] Asimakopoulos A.G., Xue J., De Carvalho B.P., Iyer A., Abualnaja K.O., Yaghmoor S.S., Kumosani T.A., Kannan K. (2016). Urinary biomarkers of exposure to 57 xenobiotics and its association with oxidative stress in a population in Jeddah, Saudi Arabia. Environ. Res..

[29] Munakata S., Ishimori K., Kitamura N., Ishikawa S., Takanami Y., Ito S. (2018). Oxidative stress responses in human bronchial epithelial cells exposed to cigarette smoke and vapor from tobacco- and nicotine-containing products. Regul. Toxicol. Pharmacol..

[30] Chao M.-R., Cooke M.S., Kuo C.-Y., Pan C.-H., Liu H.-H., Yang H.-J., Chen S.-C., Chiang Y.-C., Hu C.-W. (2018). Children are particularly vulnerable to environmental tobacco smoke exposure: Evidence from biomarkers of tobacco-specific nitrosamines, and oxidative stress. Environ. Int..

[31] Marcon A., Pesce G., Calciano L., Bellisario V., Dharmage S.C., Garcia-Aymerich J., Gislasson T., Heinrich J., Holm M., Janson C. (2018). Trends in smoking initiation in Europe over 40 years: A retrospective cohort study. PLoS ONE.

[32] Romanazzi V., Pirro V., Bellisario V., Mengozzi G., Peluso M., Pazzi M., Bugiani M., Verlato G., Bono R. (2013). 15-F2t isoprostane as biomarker of oxidative stress induced by tobacco smoke and occupational exposure to formaldehyde in workers of plastic. Sci. Total Environ. Total Environ..

[33] Migliore E., Piccioni P., Garrone G., Ciccone G., Borraccino A., Bugiani M. (2005). Changing prevalence of asthma in Turin school children between 1994 and 1999. Monaldi Arch. Chest Dis..

[34] Bono R., Bellisario V., Romanazzi V., Pirro V., Piccioni P., Pazzi M., Bugiani M., Vincenti M. (2014). Oxidative stress in adolescent passive smokers living in urban and rural environments. Int. J. Hyg. Environ. Health.

[35] Bacon D.W., Watts D.G. (1971). Estimating the Transition between Two Intersecting Straight Lines. Biometrika.

[36] Roberts L.J., Morrow J.D. (2000). Measurement of F(2)-isoprostanes as an index of oxidative stress in vivo. Free Radic. Biol. Med..

[37] Jacob K.D., Noren Hooten N., Trzeciak A.R., Evans M.K. (2013). Markers of oxidant stress that are clinically relevant in aging and age-related disease. Mech. Ageing Dev..

[38] Bono R., Tassinari R., Bellisario V., Gilli G., Pazzi M., Pirro V., Mengozzi G., Bugiani M., Piccioni P. (2015). Urban air and tobacco smoke as conditions that increase the risk of oxidative stress and respiratory response in youth. Environ. Res..

[39] Bouzid M.A., Hammouda O., Matran R., Robin S., Fabre C. (2014). Changes in oxidative stress markers and biological markers of muscle injury with aging at rest and in response to an exhaustive exercise. PLoS ONE.

[40] Heid J., Cencioni C., Ripa R., Baumgart M., Atlante S., Milano G., Scopece A., Kuenne C., Guenther S., Azzimato V. (2017). Age-dependent increase of oxidative stress regulates microRNA-29 family preserving cardiac health. Sci. Rep..

[41] Squillacioti G., Bellisario V., Grignani E., Mengozzi G., Bardaglio G., Dalmasso P., Bono R. (2019). The Asti Study: The Induction of Oxidative Stress in A Population of Children According to Their Body Composition and Passive Tobacco Smoking Exposure. Int. J. Environ. Res. Public Health.

